# Psychometric Evaluation of the Insomnia Severity Index in Chronic Disease Patients Using Three Complementary Approaches

**DOI:** 10.1002/brb3.71040

**Published:** 2025-11-10

**Authors:** Firoj Al‐Mamun, Mohammed A. Mamun, Mohammad Arif, Pronab Das, Moneerah Mohammad ALmerab, David Gozal

**Affiliations:** ^1^ CHINTA Research Bangladesh, Savar Dhaka Bangladesh; ^2^ Department of Public Health University of South Asia Dhaka Bangladesh; ^3^ Department of Ayurvedic Medicine Institute of Teaching and Research in Ayurveda Jamnagar Gujarat India; ^4^ One Health Institute Chattogram Veterinary and Animal Sciences University Chattogram Bangladesh; ^5^ Department of Psychology, College of Education and Human Development Princess Nourah Bint Abdulrahman University Riyadh Saudi Arabia; ^6^ Departments of Pediatrics and Biomedical Sciences and Office of the Dean, Joan C. Edwards School of Medicine Marshall University Huntington West Virginia USA; ^7^ School of Medicine University of Nottingham Nottingham UK

**Keywords:** Bangladesh, chronic disease, insomnia, network analysis, psychometrics, Rasch model, validation

## Abstract

**Background:**

Insomnia is highly prevalent among individuals with chronic diseases and often exacerbates disease progression while adversely impacting mental health and quality of life. However, validated tools to assess insomnia in this vulnerable population remain limited in emerging economies. This study aimed to evaluate the psychometric properties of the Bangla version of the Insomnia Severity Index (ISI) among Bangladeshi adults with chronic diseases using confirmatory factor analysis (CFA), Rasch modeling, and network analysis.

**Methods:**

A face‐to‐face survey was conducted among adults with clinically diagnosed chronic illnesses. CFA was used to test one‐, two‐, and three‐factor structures and gender‐based measurement invariance. Rasch analysis examined item fit, reliability, and response category functioning. Network analysis estimated symptom interrelations, centrality, bridge metrics, and gender‐based network invariance.

**Results:**

The two‐factor model (Night Symptoms, Daytime Impact) provided the most favorable balance between statistical fit and theoretical coherence. Although the three‐factor model yielded marginally better indices, its reliance on a single‐indicator factor and high inter‐factor correlations limited its interpretability. All items showed strong factor loadings and internal consistency. Measurement invariance was supported across gender. Rasch modeling confirmed item fit, category functioning, and reliability, though some items exhibited moderate gender‐based DIF. Network analysis identified ISI_1 (difficulty falling asleep) and ISI_6 (noticeability of sleep problems) as central symptoms, while ISI_4 (satisfaction with sleep pattern) emerged as a key bridge symptom. Predictability was high, and network structure was invariant across gender.

**Conclusion:**

The ISI‐Bangla demonstrates strong psychometric validity in a chronic disease population, supporting its use for clinical and research purposes in Bangladesh. Testing the alternative factor structures confirmed the robustness of the two‐factor specification. The integration of CFA, Rasch, and network analysis provides a comprehensive validation framework.

## Graphical abstract

The Bangla version of the ISI was validated among adults with chronic diseases using confirmatory factor analysis, Rasch modeling, and network analysis. Findings supported a two‐factor structure (nighttime symptoms, daytime impact) with strong reliability, measurement invariance across gender, and coherent symptom networks. Key central and bridging symptoms highlight clinically actionable targets, establishing the ISI‐Bangla as a robust tool for both clinical and research use in Bangladesh.

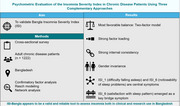

## 1 Introduction

Insomnia is a prevalent and debilitating condition characterized by difficulties in initiating and maintaining sleep and nocturnal awakenings and potentially associated with significant impairments in daytime functioning (Roth 2007). Among individuals with chronic diseases, the burden of insomnia is particularly elevated, with prevalence estimates often exceeding those reported in the general population (Ge et al. 2019; Taylor et al. 2007). For instance, individuals with specific chronic conditions report markedly higher rates of chronic insomnia compared to those without such conditions: cardiovascular disease (44.1% vs. 22.8%), cancer (41.4% vs. 24.6%), hypertension (44.0% vs. 19.3%), neurologic disease (66.7% vs. 24.3%), respiratory diseases (59.6% vs. 21.4%), urinary problems (41.5% vs. 23.3%), chronic pain (48.6% vs. 17.2%), and gastrointestinal disorders (55.4% vs. 20.0%) (Taylor et al. 2007). Sleep disturbances in this population are not only more common but also more persistent and carry an incremental clinical burden given their bidirectional associations with disease progression, quality of life, and mental health comorbidities (Buysse 2013; Riemann et al. 2015). Accurate identification and measurement of insomnia symptoms in patients with chronic diseases is therefore essential for both clinical assessments and targeted interventions.

The Insomnia Severity Index (ISI) is one of the most widely used self‐report tools for evaluating the severity and impact of insomnia symptoms. Originally developed by Bastien et al. (2001) (Bastien et al. 2001), the ISI consists of seven items assessing both nocturnal symptoms and daytime consequences of insomnia over the preceding two weeks. The scale has demonstrated robust psychometric properties across various populations, including community, clinical, and cross‐cultural samples (Bastien et al. 2001; Manzar et al. 2021; Morin et al. 2011). However, despite its global usage, the psychometric evaluation of the ISI among patients with chronic diseases (Al Maqbali et al. 2022; Jun et al. 2022), particularly in low‐ and middle‐income countries (LMICs) such as Bangladesh, remains scarce (Manzar et al. 2021).

Psychometric evaluations of the ISI have traditionally relied on classical test theory (CTT) approaches, such as confirmatory factor analysis (CFA), to determine the underlying factor structure. While several studies support the unidimensional structure of the ISI (Dragioti et al. 2015; Gerber et al. 2016; Sadeghniiat‐Haghighi et al. 2014), others propose multidimensional models (Albougami and Manzar 2019; Castronovo et al. 2016; Chen et al. 2015; Fernandez‐Mendoza et al. 2012). Inconsistencies across studies may reflect differences in cohort characteristics, linguistic and cultural factors, or methodological choices, underscoring the importance of localized validation (Manzar et al. 2021).

In recent years, modern psychometric methods such as Rasch modeling and network analysis have been increasingly employed alongside traditional techniques (i.e., CFA) to provide more nuanced evaluations of scale functioning. Rasch analysis, which is grounded in item response theory (IRT), facilitates the examination of item fit, response category functioning, and differential item functioning (DIF) across subgroups (Bond 2015). It enables the transformation of ordinal scores into interval‐level measures and supports the assessment of measurement invariance, an essential criterion for fair comparisons across demographic groups such as gender (Boone 2016). However, relatively few studies have applied Rasch modeling to the ISI. For example, a recent validation in the Korean general population supported a two‐factor structure (Chung et al. 2024), while a Bangladeshi study using a general population sample identified a unidimensional model (Mamun et al. 2021). Among clinical populations, Rasch analysis has been used to validate the ISI in cancer patients in Iran, demonstrating adequate item functioning (Lin et al. 2020).

More recently, network analysis consists of an emerging innovation in psychometric science and reconceptualizes psychological constructs by modeling symptoms as mutually interacting nodes rather than reflective indicators of a latent variable (Borsboom 2017). This approach estimates partial correlations between items to identify highly central or bridging symptoms that may serve as strategic intervention targets. Network analysis has been successfully applied to various psychological phenomena, including insomnia symptoms (Bai et al. 2021; Chen et al. 2023; Takano et al. 2023). However, applications of the ISI among chronic disease populations, especially in LMICs, are lacking.

Given the growing consensus that no single psychometric method sufficiently captures the complexities of scale performance, a multi‐method evaluation strategy is recommended for comprehensive instrument validation (Chan et al. 2014). Notably, although a previous Bangladeshi study validated the ISI during the COVID‐19 pandemic using an online general population sample (Mamun et al. 2021), that study did not target a vulnerable clinical population nor employ in‐person assessments or use network analysis. These gaps highlight the necessity of validating the ISI in a clinically relevant, medically diverse population using rigorous face‐to‐face data collection. These methodological and contextual gaps are particularly salient for chronic disease patients, whose insomnia symptoms may be shaped by intertwined biological, psychological, and social factors.

Therefore, the present study aimed to evaluate the psychometric structure of the Bangla version of the ISI in a large, diverse sample of Bangladeshi adults suffering from chronic diseases by applying three complementary approaches: (i) confirmatory factor analysis (CFA) to determine the scale's latent structure and test measurement invariance across gender; (ii) Rasch modeling to examine item‐level properties, dimensionality, and response category functioning; and (iii) network analysis to explore the symptom structure, centrality, and stability of ISI items. This interdisciplinary approach is designed to enhance behavioral assessment in clinical settings and inform tailored interventions for sleep disturbance in medically complex populations. To our knowledge, this is one of the first studies to triangulate these three psychometric frameworks for evaluating the ISI in a chronic disease population.

## 2 Methods

### 2.1 Study Design, Participants, and Procedure

This cross‐sectional investigation targeted individuals diagnosed with chronic diseases affecting various physiological systems, including but not limited to the respiratory, gastrointestinal, urinary, dermatological, reproductive, musculoskeletal, endocrine, cardiovascular, hematological, and neurological systems. Recruitment occurred across several healthcare facilities in Bangladesh, specifically the Hamdard General Hospital, the Government Unani and Ayurvedic Medical College Hospital in Dhaka, and two branches of Hamdard Waqf Bangladesh Laboratories located in Dhaka and Chittagong.

Following institutional approvals, data collection was conducted by eight trained research assistants under the supervision of a physician team leader. The data collection spanned the period from May 21 to November 10, 2024 via face‐to‐face interviews. A convenience sampling technique was employed, whereby all eligible patients attending the respective healthcare centers during the study period were invited to participate. Participants were approached during their visits, briefed on the study objectives and procedures, and informed about any potential risks or benefits as well as their rights to withdraw at any stage.

Eligibility criteria included being over 18 years of age and having a confirmed diagnosis of a chronic condition for at least three months. Those younger than 18 or with a shorter disease duration were excluded. A total of 50 respondents were excluded due to ineligibility or incomplete responses. Ultimately, the final analytical sample comprised 1,222 participants, reflecting an approximate response rate of 91%.

### 2.2 Translation Process of the ISI‐Bangla

The ISI was translated into Bangla through a forward–backward translation process to ensure linguistic and cultural relevance (Beaton et al. 2000). Three independent translators, including two public health experts and a professional linguist proficient in both English and Bangla, performed the initial forward translations. These translations were synthesized and then independently back‐translated into English by a medically literate translator and a bilingual individual unfamiliar with the original scale. All versions were reviewed by a panel of five experts, three with public health expertise (two of whom had sleep research experience) and one specializing in psychometric evaluation. The panel evaluated semantic accuracy and cultural appropriateness before finalizing the translation. The revised Bangla ISI was then pretested on a sample of 60 participants, with subsequent adjustments made based on the feedback. Data from the pre‐test group were excluded from the main study analysis.

### 2.3 Ethical Considerations

The study adhered to the ethical principles outlined in the Declaration of Helsinki (1975; revised 2024) to uphold ethical standards for human subject research. Ethical clearance was obtained from the institutional review board at CHINTA Research Bangladesh [Ref: CHINTA/2024/04‐05]. Prior to data collection, participants were given comprehensive verbal and written information regarding the study's aims, procedures, potential risks and benefits, and their rights, including the assurance of data confidentiality and the freedom to withdraw at any time. Written informed consent was obtained from all participants. Additionally, administrative approval was obtained from all participating healthcare institutions. Collected data were anonymized and stored securely, with access restricted to authorized personnel to ensure confidentiality and data protection.

### 2.4 ISI

Insomnia symptoms were assessed using the ISI (Bastien et al. 2001; Morin et al. 2011). The scale includes seven items that evaluate the severity and impact of insomnia symptoms over the past two weeks (e.g., “degree of satisfaction/dissatisfaction with current sleep pattern”). Items are rated on a 5‐point Likert scale, ranging from 0 (“not at all”) to 4 (“extremely”), yielding a total score between 0 and 28. For this study, the ISI was translated into Bangla using the previously described forward‐backward method.

### 2.5 Statistical Analysis

#### 2.5.1 CFA Analysis

CFA was performed to evaluate the factorial validity of the ISI‐Bangla. CFA was conducted using the robust weighted least squares mean and variance adjusted (WLSMV) estimator, which is recommended for ordinal data (Brown 2015; Li 2016). Competing measurement models were tested, including a unidimensional (one‐factor) model and a theoretically supported two‐factor model representing night symptom and daytime impact dimensions. The three‐factor model was specified as Night Symptoms (ISI_1‐3), Satisfaction with sleep (ISI_4), and Daytime impact (ISI_5‐7), in line with prior research (Chen et al. 2015; Bastien et al. 2001). To improve model fit while maintaining theoretical coherence, modification indices were examined. Based on both empirical evidence and conceptual justification, residual covariances were allowed between ISI_1 and ISI_2 (reflecting overlapping assessment of difficulty falling asleep) and between ISI_6 and ISI_7 (both measuring daytime consequences of poor sleep). Additionally, the Night and Day factors were allowed to correlate. For the three‐factor model, the three latent factors were allowed to covary (i.e., their covariances were estimated), consistent with the two‐factor specification. Factor variances were fixed to 1, and the Satisfaction factor (ISI_4) was identified by fixing its loading to 1 and residual variance to 0. Model fit was assessed using multiple goodness‐of‐fit indices: Comparative Fit Index (CFI), Tucker‐Lewis Index (TLI), Root Mean Square Error of Approximation (RMSEA), and Standardized Root Mean Square Residual (SRMR). Acceptable model fit was defined as CFI and TLI > 0.90, RMSEA < 0.06, and SRMR < 0.08 (Brown 2015; Hu and Bentler 1999).

Measurement invariance across gender was examined via multi‐group CFA by sequentially testing configural, metric, and scalar invariance. Invariance was evaluated by changes in fit indices, with thresholds of ΔCFI < 0.01, ΔRMSEA < 0.015, and ΔSRMR < 0.03 (Chen 2007). To assess reliability and convergent validity, standardized factor loadings, Average Variance Extracted (AVE), Composite Reliability (CR), and internal consistency indices (Cronbach's alpha, ordinal alpha, and McDonald's omega) were computed (Cheung et al. 2024). All statistical analyses were conducted in R version 4.4.1 using the *lavaan* and *semTools* packages.

#### 2.5.2 Rasch Analysis

Rasch analysis was performed to examine the psychometric properties of the ISI‐Bangla. Given the polytomous nature of the ISI items, the Partial Credit Model (PCM) was applied using conditional maximum likelihood estimation (Masters 1982). Analyses were carried out in R using the *TAM* and *eRm* packages. Prior to model estimation, response category functioning was evaluated. Several ISI items displayed disordered thresholds in the original 5‐point scale (0–4), indicating that respondents could not consistently differentiate between adjacent categories. Based on empirical response distributions and theoretical interpretability, categories were collapsed into a 4‐point scale (0–3) to restore monotonic threshold ordering (Bond 2015). The revised response structure demonstrated properly ordered thresholds across all items.

Dimensionality was assessed via principal component analysis (PCA) of model residuals. An eigenvalue below 2.0 for the first residual component supported unidimensionality. Residual correlations between item pairs were evaluated using Yen's Q3 statistic, with values > 0.20 above the average suggesting local dependence (Christensen et al. 2017; Yen 1993). Item fit was evaluated using infit and outfit mean square (MnSq) statistics, with acceptable values ranging from 0.7 to 1.3 (Bond 2015). Items outside this range were examined for potential misfit. Differential item functioning (DIF) was analyzed by gender using Andersen's Likelihood Ratio (LR) Test and effect size estimates. A DIF contrast ≥ 0.50 logits was considered evidence of meaningful DIF (Johansson et al. 2023). Reliability and separation indices were computed for persons and items. A person separation index (PSI) > 2.0 indicates the scale can distinguish between at least three trait levels, while item separation reflects the replicability of item ordering. Person reliability was estimated via Expected A Posteriori (EAP). All Rasch analyses were performed in R version 4.4.1 using eRm (v1.0‐2), TAM (v4.1‐2), and supporting packages such as psych, polycor, and ggplot2 for diagnostics and visualizations.

#### 2.5.3 Network Analysis

A network analysis was conducted to explore the structure and connectivity of insomnia symptoms in the ISI‐Bangla. The network was estimated using the Extended Bayesian Information Criterion Graphical Lasso (EBICglasso) algorithm via the *estimateNetwork()* function in the *bootnet* package, which is suitable for sparse regularized estimation in ordinal data (Epskamp et al. 2018). The network was visualized using the *qgraph* package with the Fruchterman–Reingold layout, where nodes represented individual ISI items and edges indicated regularized partial correlations (Epskamp et al. 2012). Positive edges were shown in blue and negative edges in red, with edge thickness reflecting the strength of association. To quantify node importance, centrality indices—strength, closeness, and betweenness—were computed using centrality() and visualized with *centralityPlot()*. Predictability, defined as the proportion of variance in a node explained by its neighbors, was computed using the *mgm* package (Haslbeck and Waldorp 2020). Community detection was conducted using the Spinglass algorithm implemented in the igraph package to identify symptom clusters. To assess cross‐cluster connectivity, bridge centrality metrics, including bridge strength, closeness, betweenness, and expected influence, were computed using the *networktools* package (Jones et al. 2021). Stability and accuracy of the network were evaluated using nonparametric and case‐dropping bootstrapping (1,000 iterations) via the *bootnet* package. The edge weight accuracy was assessed by generating 95% confidence intervals, while the centrality stability was evaluated using the Correlation Stability (CS) coefficient, with CS ≥ 0.70 considered excellent and > 0.50 acceptable (Epskamp et al. 2018). A Network Comparison Test (NCT) was performed to examine gender‐based differences using the *NetworkComparisonTest* package (van Borkulo et al. 2022). The test assessed differences in (1) overall network structure and (2) global strength (total connectivity). Significance was evaluated via 1000 permutations. All analyses were conducted in R version 4.4.1 using RStudio (2023.06.1).

## 3 Results

### 3.1 CFA Analysis

#### 3.1.1 Factor Structure of ISI‐Bangla

To examine the latent structure of the ISI‐Bangla, CFA was conducted to compare a one‐factor and a theoretically supported two‐factor model and a three‐factor model (Table 1). The one‐factor model demonstrated poor fit, with robust CFI (0.764) and TLI (0.646) values substantially below the recommended cutoff of 0.90, a robust RMSEA of 0.350 greatly exceeding the accepted threshold of 0.08, and an SRMR of 0.098 indicating poor fit. In contrast, the two‐factor solution showed a much stronger fit to the data, with a robust CFI of 0.955 (good fit), a robust TLI of 0.914 (acceptable fit), and an SRMR of 0.040 (excellent fit). However, the robust RMSEA for the two‐factor model remained elevated at 0.173, suggesting some residual model misfit. The three‐factor solution (Nighttime symptoms: ISI_1–3; Satisfaction: ISI_4; Daytime impact: ISI_5–7) demonstrated further improvement in global fit indices, with robust CFI = 0.981 and TLI = 0.960 (both excellent), SRMR = 0.021 (excellent), though the robust RMSEA remained above acceptable thresholds at 0.117. All standardized loadings were strong (0.83–0.96). However, correlations among the three latent factors were high (r = 0.76–0.82), and the Satisfaction factor was represented by a single item, limiting its interpretability. Thus, while the three‐factor model showed improved fit relative to the one‐ and two‐factor solutions, the two‐factor structure was retained as the most parsimonious and theoretically interpretable representation of the ISI‐Bangla (Table 1).

**TABLE 1:** Comparing CFA for ISI‐Bangla: One, two, and three‐factor model.

Fit index	Robust value	Cutoff	Interpretation
One‐factor model			
CFI	0.764	> 0.9	Not acceptable
TLI	0.646	> 0.9	Not acceptable
RMSEA	0.350	< 0.06	Not acceptable
SRMR	0.098	< 0.08	Poor
Two‐factor model			
CFI	0.955	> 0.9	Good
TLI	0.914	> 0.9	Acceptable
RMSEA	0.173	< 0.06	Poor
SRMR	0.040	< 0.08	Excellent
Three‐factor model			
CFI	0.981	> 0.9	Excellent
TLI	0.960	> 0.9	Excellent
RMSEA	0.117	< 0.06	Poor
SRMR	0.021	< 0.08	Excellent

#### 3.1.2 Item‐Level and Reliability Indices

As summarized in Table 2, all ISI items exhibited strong standardized factor loadings (> 0.80), indicating that each item is a robust indicator of its underlying latent construct, Night Symptom (items 1–4) or Daytime Impact (items 5–7). The average variance extracted (AVE) was 0.761 for Night Symptom and 0.829 for Daytime Impact, exceeding the recommended minimum of 0.50 and reflecting good convergent validity. Composite reliability (CR) values were also high for both factors (0.927 and 0.935, respectively), supporting the internal consistency of the subscales. Cronbach's alpha and ordinal alpha values (α/α_or_𝒹) were both above 0.88, while McDonald's omega coefficients were 0.864 (night symptom) and 0.912 (daytime impact), indicating excellent reliability for both subscales (Cheung et al. 2024).

**TABLE 2:** Item‐level and reliability analysis of the ISI‐Bangla.

ISI item	Latent factor	Standardized loading (CFA)	AVE	CR	*α*/ *α*_or_𝒹	*ω*
ISI_1 – Difficulty falling asleep	Night Symptom	0.814	0.761	0.927	0.903/0.935	0.864
ISI_2 – Difficulty staying asleep	Night Symptom	0.836				
ISI_3 – Problem waking up too early	Night Symptom	0.933				
ISI_4 – Satisfaction with current sleep pattern	Night Symptom	0.901				
ISI_5 – Interference with daily functioning	Daytime Impact	0.836	0.829	0.935	0.889/0.929	0.912
ISI_6 – Noticeability of sleep problems by others	Daytime Impact	0.952				
ISI_7 – Worry/distress about current sleep problem	Daytime Impact	0.939				

*Note*: AVE, CR, *α*, *α*_or_𝒹, and *ω* are reported at the factor level, not the item level.

**Abbreviations**: *α*, Cronbach's alpha assumes continuous indicators; α_or_𝒹, ordinal alpha based on polychoric correlations, recommended for ordinal data; AVE, average variance extracted; CFA, confirmatory factor analysis; CR, composite reliability; *ω*, McDonald's omega, a more robust estimate of internal consistency.

For the three‐factor specification (night symptom, satisfaction, daytime impact), reliability indices were highly similar: AVE = 0.79–0.83 and CR = 0.92–0.94, with α/α_or_𝒹 > 0.88 and ω ≈ 0.85–0.91. However, the satisfaction factor was a single‐indicator construct (ISI_4), yielding artificially perfect AVE and CR values (1.00). Full item‐level and reliability results for the three‐factor model are presented in Supplementary Table S1.

#### 3.1.3 Measurement Invariance Across Gender

Measurement invariance was examined using a multi‐group CFA framework across gender (Table 3). The configural model (i.e., unconstrained baseline model) provided a good fit (CFI = 0.935; RMSEA = 0.210; SRMR = 0.043), indicating that the basic factor structure of the ISI‐Bangla is similar across male and female respondents. Imposing metric invariance (constraining factor loadings to be equal) resulted in only minimal changes in fit indices (ΔCFI = −0.007, ΔRMSEA = −0.011, ΔSRMR = +0.006), all well within the recommended thresholds (i.e., ΔCFI < 0.01, ΔRMSEA < 0.015, ΔSRMR < 0.03) (Chen 2007), thus supporting invariance of item‐factor relationships across gender. Due to limitations of the WLSMV estimator, robust CFI and RMSEA values for the scalar model could not be computed, which is a known methodological issue (Xia and Yang 2019). Nonetheless, the SRMR value for the scalar model (0.044) and its small change relative to the metric model (ΔSRMR = −0.005) suggest no substantial worsening of fit, lending further support to partial scalar invariance.

**TABLE 3:** Measurement invariances across gender for the two‐factor ISI‐Bangla.

Invariance level	CFI	ΔCFI	RMSEA	ΔRMSEA	SRMR	ΔSRMR
Configural	0.935	—	0.210	—	0.043	—
Metric	0.928	−0.007	0.199	−0.011	0.049	+0.006
Scalar	NA	NA	NA	NA	0.044	−0.005

*Note*: Scalar invariance could not be fully assessed due to missing robust indices under WLSMV, which is a known limitation.

### 3.2 Rasch Analysis

#### 3.2.1 Distribution of Response Categories

**FIGURE 1:**
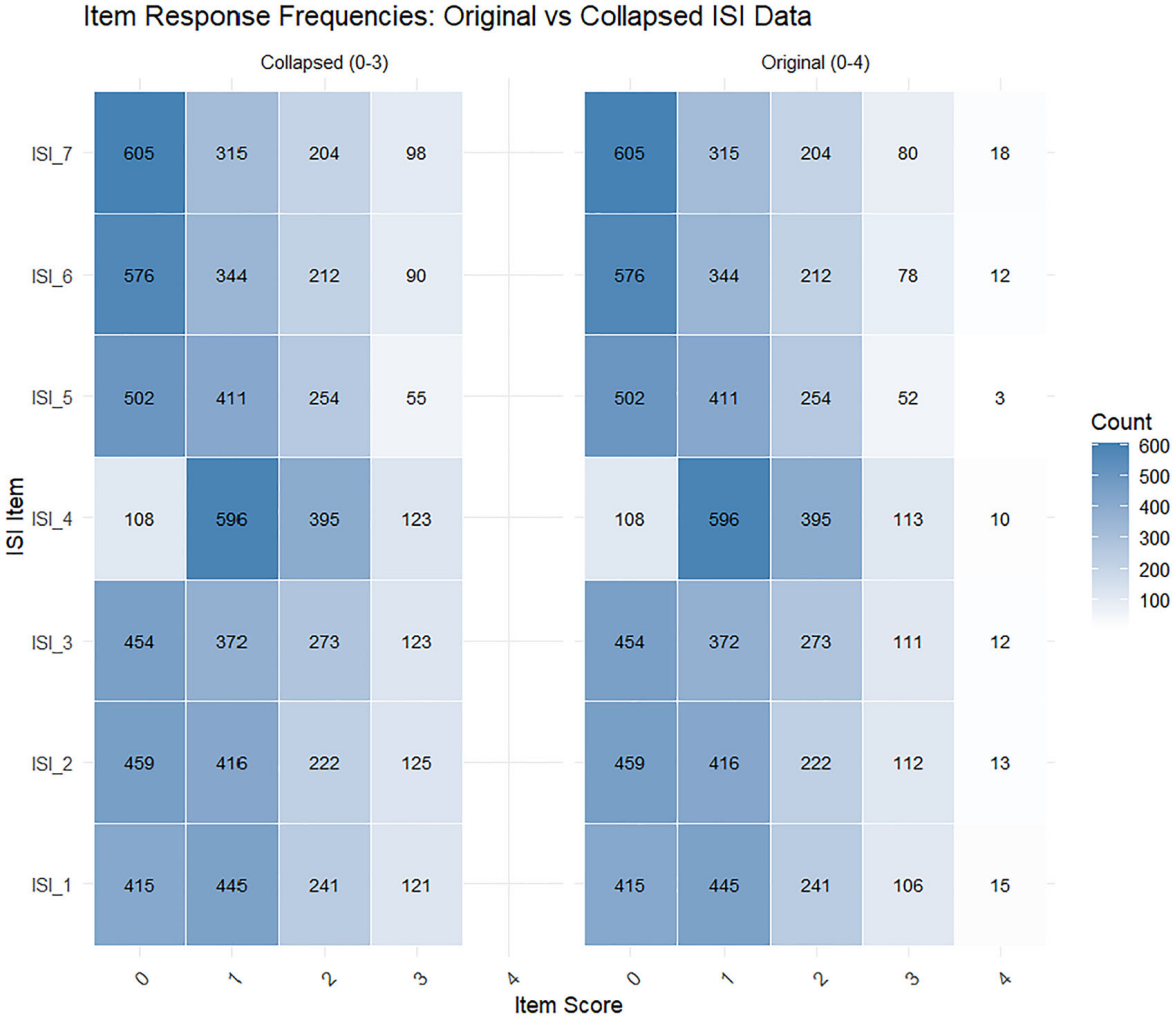
Item response frequencies for original (0–4) vs. collapsed (0–3) ISI categories.

Figure 1 compares the distribution of item responses before and after collapsing sparse upper categories. In the original ISI format (0–4 scale), response option 4 (very severe) was infrequently endorsed across all items, particularly for ISI_5 to ISI_7, raising concerns about category underutilization. After collapsing the top two categories into a single “3” category, the collapsed version (0–3) showed improved distributional balance across response options. This collapsing procedure was guided by recommendations from Linacre (2002) (Linacre 2002), where response categories with very low frequencies (fewer than 10 observations) should be merged to ensure model estimation stability and interpretability in Rasch modeling (Quan and Wang 2025). As shown, the collapsed structure avoided extreme sparsity and preserved the ordinal nature of responses, justifying its use for subsequent Rasch modeling.

#### 3.2.2 Item Characteristic Curves

**FIGURE 2:**
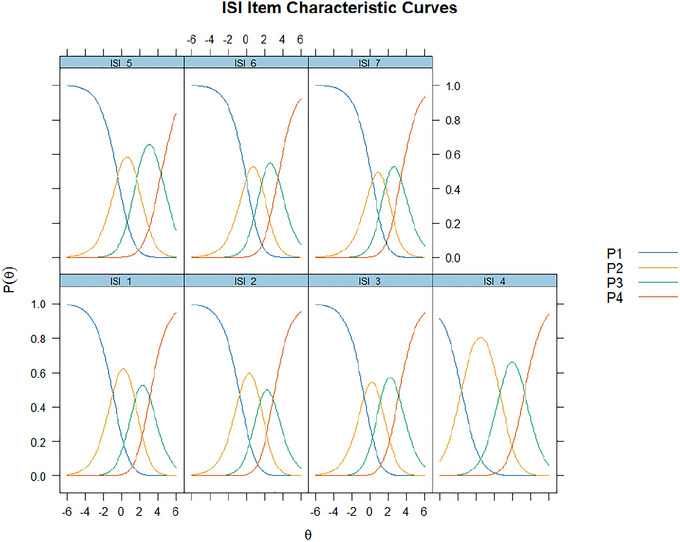
Item characteristic curves (Rasch PCM; collapsed 0–3 categories).

Figure 2 displays the Item Characteristic Curves (ICCs) for each ISI item under the Rasch Partial Credit Model using the collapsed 4‐point scale (0–3). Each curve represents the probability of endorsing a particular response category (P1–P4) as a function of the latent trait (*θ*). Across all items, the response categories showed appropriate monotonic ordering and threshold spacing, suggesting that the categories were functioning as intended. The curves demonstrate clear separation between adjacent categories, indicating that each category captures a distinct range of the underlying insomnia severity continuum. Some minor overlaps were observed between adjacent categories in a few items (e.g., ISI_4 and ISI_5), but these were within acceptable bounds and did not compromise interpretability. Overall, the ICCs support the category structure and discrimination of the items in measuring insomnia severity among patients with chronic diseases.

#### 3.2.3 Scale Dimensionality

A PCA conducted on the polychoric correlation matrix of the ISI‐Bangla items supported a two‐factor solution, aligning with the hypothesized dimensional structure. The first and second components yielded eigenvalues of 3.08 and 3.04, respectively, both surpassing the Kaiser criterion of 1, indicating that each factor meaningfully contributes to explaining item variance. The first component (RC1), which accounted for 44% of the total variance, was primarily defined by the Daytime Impact items (ISI_5 to ISI_7). The second component (RC2) explained an additional 43% of the variance and corresponded to Nighttime Symptom items (ISI_1 to ISI_3). Notably, ISI_4 showed substantial cross‐loadings on both components, suggesting it may function as a bridging item between the two latent dimensions. All items demonstrated high communalities (*h* ^2^ ≥ 0.81), and the model exhibited excellent overall fit (RMSR = 0.03). These findings provide strong empirical support for a multidimensional structure and reinforce the appropriateness of modeling the ISI‐Bangla using a two‐factor Rasch framework. See Supplementary Table S2 for more information on the residual matrix correlation.

#### 3.2.4 Rasch Analysis of the ISI Bangla

Table 4 presents the item‐level psychometric properties of the ISI‐Bangla based on the PCM, examining item difficulties, fit statistics, differential item functioning (DIF) by gender, and reliability and separation indices for both persons and items.

**TABLE 4:** Rasch analysis (Partial Credit Model) for ISI‐Bangla.

Item	Difficulty	SE	Infit MnSq	Outfit MnSq	DIF effect size	Person		Item	
						Separation	Reliability	Separation	Reliability
ISI_1	1.33	0.10	0.86	2.64	0.29	2.54	0.87	6.08	0.97
ISI_2	1.44	0.10	0.89	1.05	0.10				
ISI_3	1.33	0.10	0.86	3.07	0.40				
ISI_4	0.08	0.11	1.02	2.15	0.38				
ISI_5	2.14	0.11	0.98	2.87	0.33	1.89	0.81	0.39	0.13
ISI_6	2.06	0.11	0.86	0.59	0.15				
ISI_7	2.07	0.10	0.92	0.94	0.30				

*Note*: Difficulties represent average item thresholds from the Partial Credit Model (PCM). SEs reflect the average standard error across thresholds for each item.

DIF effect size: (Female − Male).

The average item difficulties ranged from 0.08 logits (ISI_4; satisfaction with sleep pattern) to 2.14 logits (ISI_5; interference with daily functioning), indicating that the ISI‐Bangla spans a wide range of insomnia severity. Night symptom items (ISI_1–4) were relatively easier, while daytime impact items (ISI_5–7) were more difficult, consistent with their expected placement along the latent continuum of insomnia severity. Standard errors (SEs) were uniformly low (0.10–0.11), reflecting precise estimation of item locations.

MnSq values for all items fell within acceptable bounds (0.86 to 1.02), suggesting that item responses conformed well to model expectations and contributed meaningfully to the measurement construct. However, several items showed high outfit MnSq values, particularly ISI_1 (2.64), ISI_3 (3.07), ISI_4 (2.15), and ISI_5 (2.87), indicating potential response anomalies among respondents with extreme trait levels. While not uncommon in patient samples, these values should be interpreted with caution and monitored in future applications (Bond 2015; Johansson et al. 2023).

The Andersen LR‐test by gender revealed a significant result for both the night items (*p* = 0.001) and the day items (*p* = 0.09), suggesting an item‐based DIF test. Gender‐based DIF was evaluated by computing the difference in item difficulty between females and males. While all DIF effect sizes remained below the 0.50‐logit threshold commonly considered indicative of large DIF (Johansson et al. 2023), moderate DIF was observed for ISI_3 (0.40), ISI_4 (0.38), and ISI_5 (0.33). These results suggest that these items may function slightly differently across gender groups, although the magnitude is unlikely to compromise group comparisons at the scale level.

The person separation index was 2.54 for night symptoms and 1.89 for daytime impact, corresponding to three and two statistically distinguishable levels of person ability, respectively. Person reliability values were acceptable for both subscales (0.87 for night symptoms and 0.81 for daytime impact), indicating sufficient internal consistency for distinguishing between individuals. Item reliability for the night symptom subscale was excellent (0.97; separation = 6.08), demonstrating high confidence in the replicability of item hierarchy. In contrast, item reliability was poor for the daytime impact subscale (0.13; separation = 0.39), likely due to limited item variability or insufficient trait dispersion in the sample, which suggests caution when interpreting item ranks in this subscale and highlights the need for future item refinement.

### 3.3 Network Analysis

#### 3.3.1 Network Structure of the Insomnia Severity Index Bangla

**FIGURE 3:**
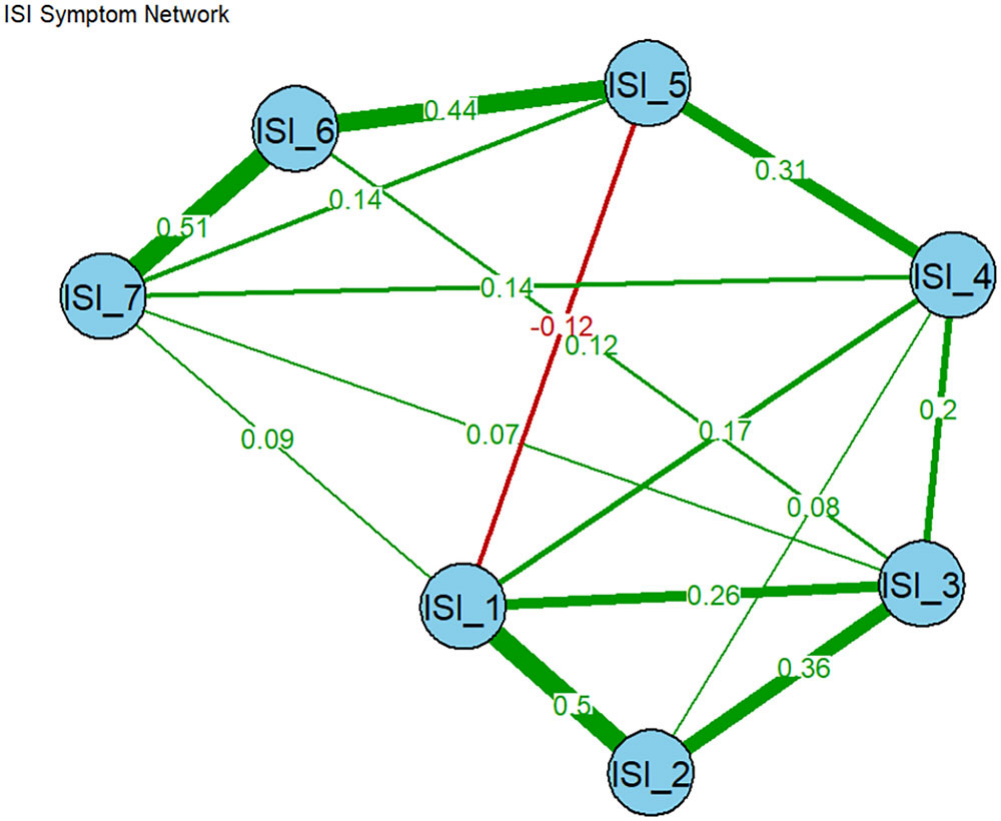
Network structure of ISI.

The network analysis of the ISI‐Bangla items revealed a well‐connected symptom structure, estimated using the EBICglasso algorithm with polychoric correlations appropriate for ordinal data (Figure 3). The strongest connection in the network was observed between ISI_6 (“Noticeability of sleep problems by others”) and ISI_7 (“Worry/distress about current sleep problems”), with an edge weight of 0.51. This strong association highlights a perceptual‐emotional symptom cluster, suggesting that individuals who feel their sleep problems are noticeable to others also experience heightened distress or worry about these issues. Among the nighttime symptom domain, ISI_1 (“Difficulty falling asleep”) and ISI_2 (“Difficulty staying asleep”) exhibited a robust association (0.50), indicating a core night‐onset maintenance dyad. Furthermore, ISI_2 was strongly connected with ISI_3 (“Problem waking up too early”; 0.36), forming a triadic core of nocturnal disturbance.

Cross‐domain connections were most prominent between ISI_4 (“Satisfaction with current sleep pattern”) and ISI_5 (“Interference with daily functioning”; 0.31), underscoring the influence of subjective sleep satisfaction on daytime performance. Notably, ISI_5 was also positively connected to ISI_6 (0.44) and ISI_7 (0.14), situating it as a potential bridge between night‐related complaints and daytime consequences.

The network was overall positively connected, with many medium‐strength associations (e.g., ISI_3–ISI_4: 0.20, ISI_1–ISI_3: 0.26), supporting the coherent structure of the ISI as a scale measuring a unified yet multidimensional construct. The presence of denser edges among night symptoms (ISI_1 to ISI_4) and among daytime and distress‐related symptoms (ISI_5 to ISI_7) further supports the conceptual two‐factor model established in the CFA and Rasch analysis (See Supplementary Table S3 for more information).

#### 3.3.2 Centrality and Predictability of ISI‐Bangla Items

**FIGURE 4:**
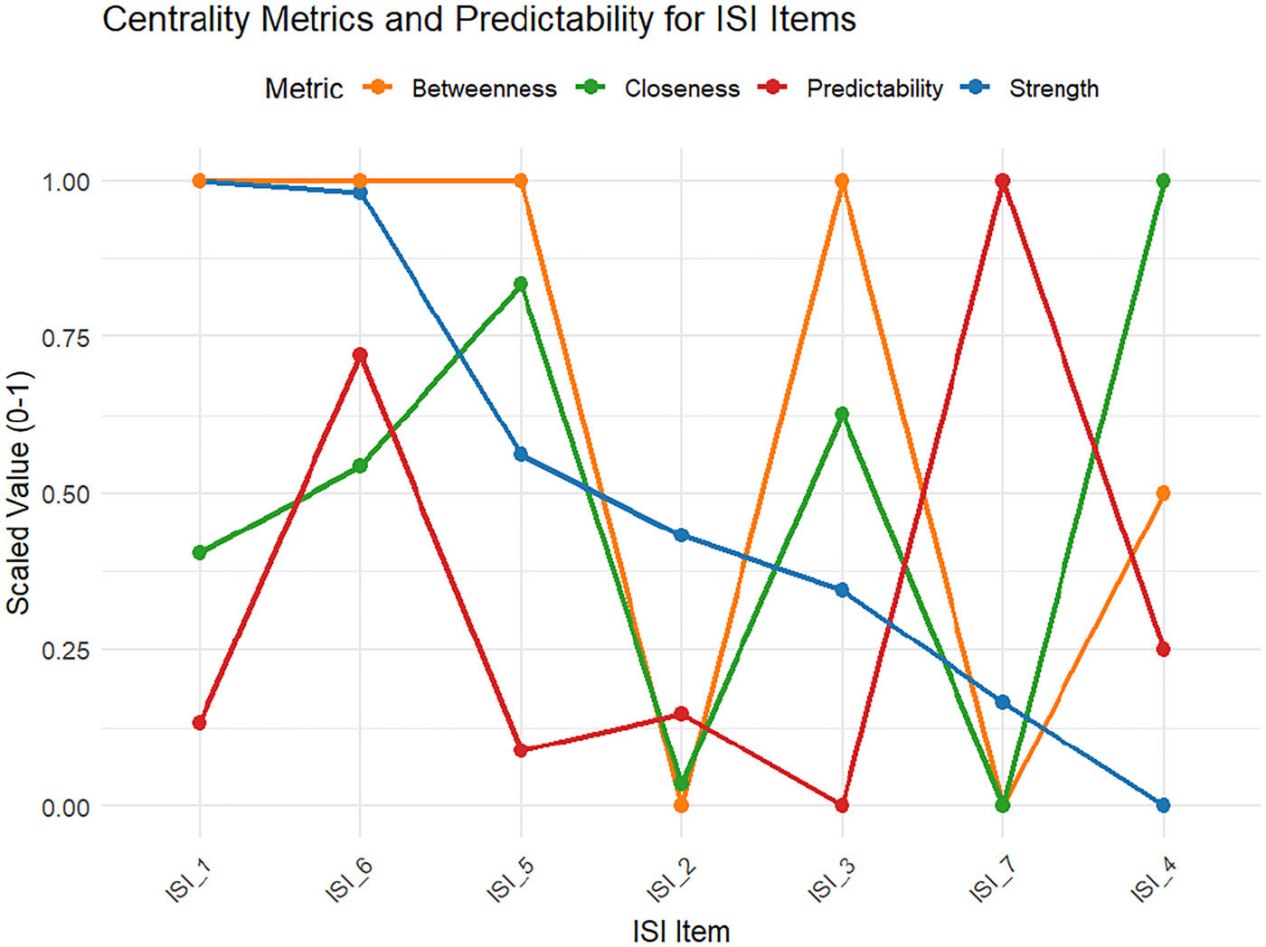
Centrality metrics and predictability for ISI items.

Figure 4 presents the centrality and predictability indices of the ISI‐Bangla items derived from the symptom network (see Supplementary Table S4 for more information).

Strength centrality, which reflects the sum of absolute edge weights connected to each node, was highest for ISI_1 (“Difficulty falling asleep”) and ISI_6 (“Noticeability of sleep problems by others”), both with scaled strength values near 1.0. These symptoms are the most connected in the network and may act as key targets for intervention. In contrast, ISI_4 (“Satisfaction with current sleep pattern”) had the lowest strength, suggesting a more peripheral role.

Betweenness centrality, which captures how often a node lies on the shortest path between other pairs of nodes, was maximized for ISI_1, ISI_3, ISI_5, and ISI_6 (value = 4). These items function as bridges facilitating symptom flow, implying that they may help transmit activation between different symptom clusters.

Closeness centrality, a measure of average distance to all other nodes, was highest for ISI_4 and ISI_5, indicating that changes in these symptoms could quickly influence the rest of the network. Interestingly, despite low strength, ISI_4 showed the highest closeness, pointing to a unique structural role that may not rely on strong direct connections.

Predictability, or node‐wise R^2^, assesses how well a given symptom is explained by its immediate neighbors. ISI_7 (“Worry/distress about current sleep problem”) and ISI_6 showed the highest predictability values (0.77 and 0.76, respectively), implying that these nodes are strongly determined by other symptoms in the network. In contrast, ISI_3 had the lowest predictability (≈0.71), indicating a somewhat more autonomous role within the network.

#### 3.3.3 Community Detection of ISI‐Bangla Items

**FIGURE 5:**
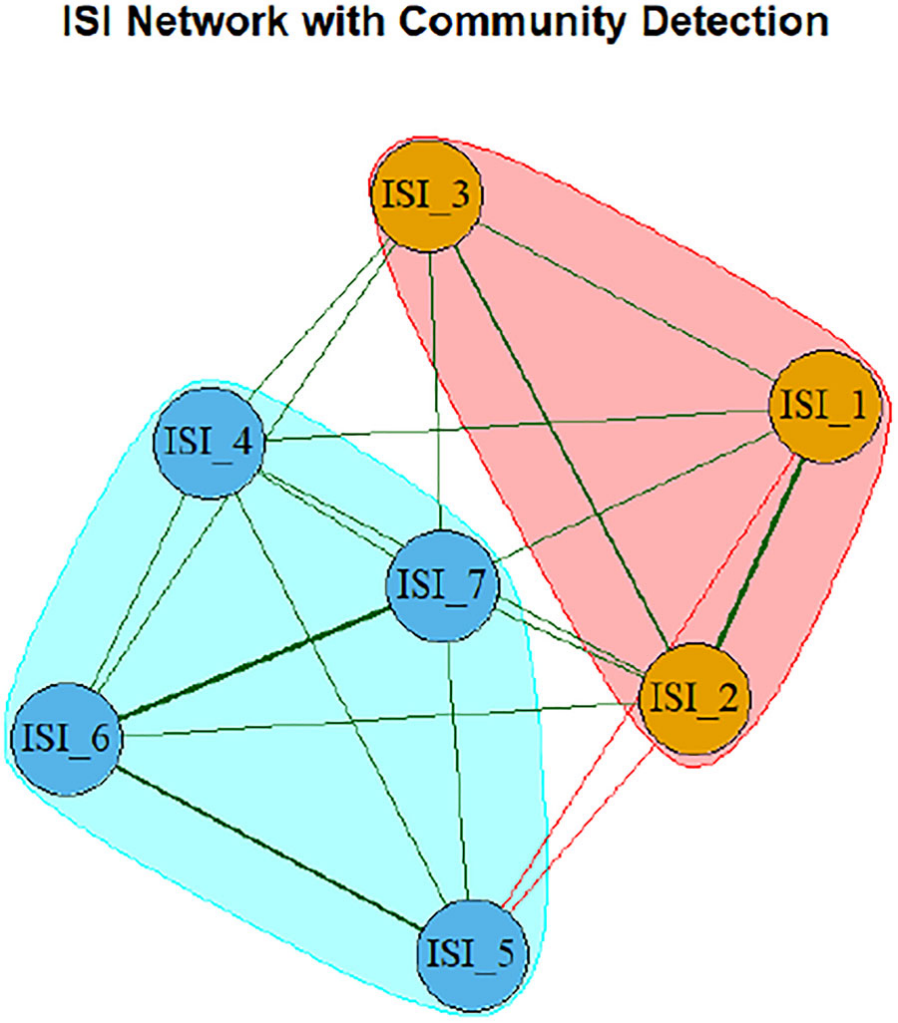
Community detection of ISI items (Spinglass algorithm).

Figure 5 illustrates the modular structure of the ISI‐Bangla symptom network using a spinglass community detection algorithm. The network was partitioned into two distinct communities, supporting the conceptual bifactorial structure of the scale. One community (highlighted in orange) encompassed ISI_1 (“Difficulty falling asleep”), ISI_2 (“Difficulty staying asleep”), and ISI_3 (“Problem waking too early”), representing Nighttime Symptoms. The second community (highlighted in blue) included ISI_4 (“Satisfaction with sleep pattern”), ISI_5 (“Interference with daily functioning”), ISI_6 (“Noticeability of sleep problems”), and ISI_7 (“Worry/distress about current sleep problem”), aligning with the Daytime Impact construct.

#### 3.3.4 Bridge Centrality Analysis

**FIGURE 6:**
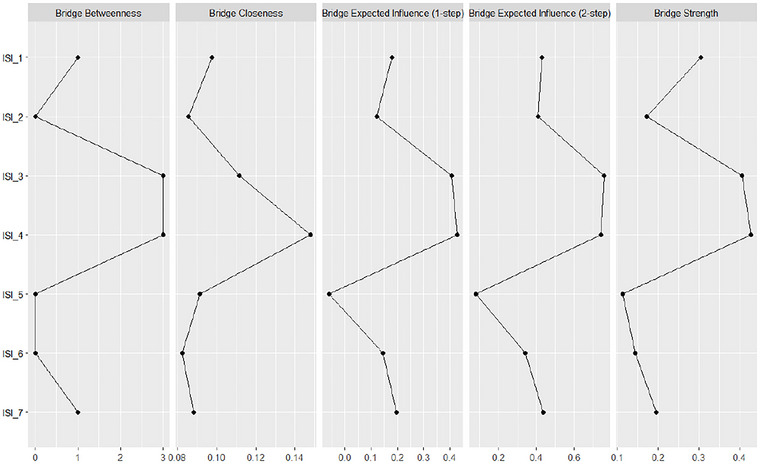
Bridge centrality metrics for ISI items.

To identify symptoms that function as bridges between distinct symptom clusters in the insomnia network, we examined several bridge centrality metrics: bridge betweenness, bridge closeness, bridge strength, and bridge expected influence (one‐step and two‐step). As shown in Figure 6, ISI_4 (“Satisfaction with current sleep pattern”) exhibited the highest bridge centrality across nearly all indices, including bridge closeness, bridge expected influence (both one‐step and two‐step), and bridge strength. This suggests that ISI_4 may play a pivotal integrative role, linking symptoms related to nighttime sleep difficulties and daytime functional impairment (**see** Supplementary Table S5 for more information).

#### 3.3.5 Network Stability and Accuracy

The robustness of the network model was evaluated using case‐dropping subset bootstrap procedures (Epskamp et al. 2018). The correlation stability coefficient (CS‐coefficient) quantifies the maximum proportion of cases that can be dropped while maintaining a correlation of at least 0.7 with the original sample estimate in 95% of the bootstrapped samples. A CS coefficient above 0.25 is considered acceptable, while a value above 0.50 is preferable for stable estimation.

The edge weight CS coefficient was 0.75, indicating excellent stability of the edge weight estimates. In contrast, the CS‐coefficient for node strength was 0.36, which meets the minimum threshold for interpretability (≥ 0.25), suggesting moderate stability of strength centrality indices. These values support the reliability of the network structure and justify cautious interpretation of strength‐based centrality metrics.

**FIGURE 7:**
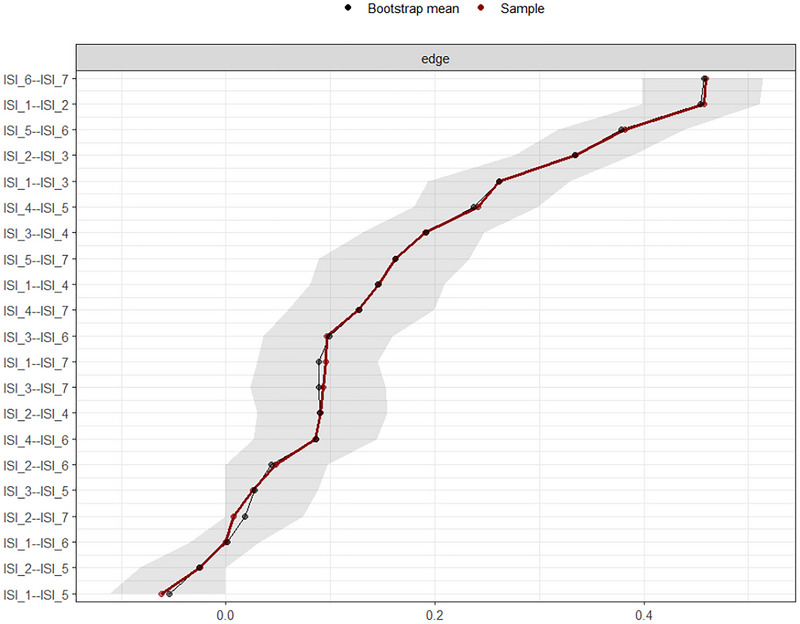
Edge weight accuracy (non‐parametric bootstrap) for ISI items **[** *Note*: The gray area represents the bootstrapped confidence intervals (CIs). The horizontal axis represents the edge weight, while the vertical axis represents the edges between each pair of nodes.

**FIGURE 8:**
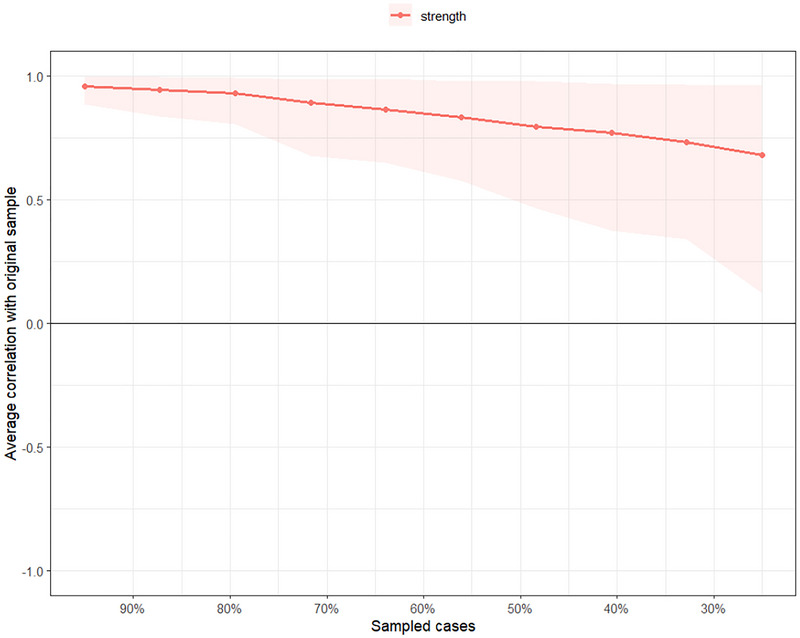
Network stability plot (e.g., strength) for ISI items by case‐dropping subset bootstrap.

Together with the narrow bootstrapped confidence intervals (**see** Figure 7) and the stability plot (Figure 8), these results indicate that the estimated network of ISI symptoms is robust to sub‐sampling and can be considered a reliable representation of symptom relationships in this dataset.

#### 3.3.6 Network Invariance Across Gender

To examine potential gender‐based differences in the structure and connectivity of insomnia symptoms, a Network Comparison Test (NCT) was conducted using the NetworkComparisonTest package in R (van Borkulo et al. 2022). The NCT assesses both network structure invariance (M‐statistic) and global strength invariance (S‐statistic) between two groups.

Results indicated no significant difference in network structure between males and females (M = 0.13, p = 0.51), suggesting that the configuration of symptom‐to‐symptom associations did not vary across gender groups. Similarly, global strength—the overall level of connectivity in the network did not differ significantly between males (S = 3.42) and females (S = 3.29), with a non‐significant test statistic (S = 0.12, p = 0.52).

These findings indicate network invariance across gender, implying that insomnia symptoms are similarly interconnected for both male and female participants in the present sample. This supports the generalizability of the network structure across gender and suggests that interventions targeting core symptoms may be effective across genders.

## 4 Discussion

### 4.1 Summary of Key Findings

This study represents the first comprehensive psychometric evaluation of the Bangla version of the ISI among a large sample of adults with chronic diseases in Bangladesh, employing a triangulated approach encompassing CFA, Rasch modeling, and network analysis. The findings provide converging evidence supporting the structural validity, item functioning, and clinical relevance of the ISI in this population.

The CFA supported a two‐factor model of the ISI‐Bangla, comprising Night Symptoms (items 1–4) and Daytime Impact (items 5–7), which provided a substantially better fit to the data compared to the unidimensional model. A three‐factor model (Night Symptom, Satisfaction, Daytime Distress) was also tested and demonstrated improved fit indices, with strong item loadings and good reliability. However, this model included a single‐indicator factor (Satisfaction) and yielded very high correlations between factors, limiting its interpretability. All items exhibited high factor loadings (> 0.80), excellent internal consistency (CR > 0.92), and satisfactory convergent validity (AVE > 0.75). Measurement invariance analysis confirmed configural, metric, and partial scalar invariance across gender, supporting the comparability of ISI scores between male and female respondents. The Rasch Partial Credit Model validated the psychometric performance of the ISI‐Bangla, especially after collapsing sparse response categories. All items demonstrated acceptable fit, though some outfit values were elevated for respondents at extreme trait levels. Dimensionality assessment via PCA supported a two‐factor structure, consistent with CFA results. The scale showed good person reliability for both subscales (Night: 0.87; Day: 0.81), though item reliability was poor for the Daytime Impact subscale (0.13), indicating potential measurement limitations. Moderate gender‐based DIF was observed for several items but did not exceed critical thresholds. Network analysis revealed a coherent and positively connected symptom structure with two main communities: night‐related symptoms (ISI_1–3) and daytime/distress symptoms (ISI_4–7). ISI_1 (“Difficulty falling asleep”) and ISI_6 (“Noticeability of sleep problems”) were the most central nodes, while ISI_4 (“Satisfaction with current sleep pattern”) showed the highest bridge centrality, linking night and day symptom clusters. Predictability values for most items were high (R^2^ > 0.72), and bootstrapped stability metrics supported the robustness of both edge and centrality estimates. Network comparison tests found no gender‐based differences in structure or global strength, confirming invariance across male and female groups. These findings support the ISI‐Bangla as a reliable and valid instrument for assessing insomnia symptoms among patients with chronic diseases in Bangladesh. The convergence of results across complementary psychometric approaches enhances confidence in the instrument's clinical and research utility.

### 4.2 CFA Findings in Context

The CFA results of the present study provide strong support for a two‐factor structure of the Bangla version of the ISI, consistent with the theoretical conceptualization distinguishing between *nighttime symptoms* (items 1–4) and *daytime impact* (items 5–7). This structure demonstrated superior model fit compared to the unidimensional solution, as evidenced by robust CFI and TLI values exceeding 0.90 and a markedly lower SRMR. Although the RMSEA for the two‐factor model was elevated, similar discrepancies in RMSEA performance have been reported in prior studies using ordinal data and WLSMV estimators, which tend to inflate this index under certain conditions (Li 2016; Xia and Yang 2019). A three‐factor model was additionally examined. While this solution showed better global fit, it relied on a single‐indicator factor and produced high latent correlations, raising concerns about discriminant validity. Thus, we retained the two‐factor specification as the most parsimonious and theoretically coherent structure.

These findings align closely with earlier validations of the ISI across various cultural contexts. For example, a two‐factor solution in the Persian version among clinic patients was identified (Sadeghniiat‐Haghighi et al. 2014), while a similar structure was reported in the Korean general population using both CFA and item response theory approaches (Chung et al. 2024); among chronic disease patients in Saudi Arabia (Al Maqbali et al. 2022); and among Saudi nurses (Albougami and Manzar 2019). These convergences suggest that the bidimensional architecture of the ISI, capturing both nocturnal complaints and their functional consequences, is robust across diverse populations. Importantly, the high standardized factor loadings (all > 0.80) observed in this study are in line with previous CFA studies (Al Maqbali et al. 2022; Albougami and Manzar 2019; Gerber et al. 2016), indicating that the Bangla ISI items are strong indicators of their respective latent constructs. The findings also suggest excellent internal consistency and convergent validity, consistent with previous findings (Fernandez‐Mendoza et al. 2012; Gerber et al. 2016; Jun et al. 2022; Mamun et al. 2021). Furthermore, the evidence of measurement invariance across gender enhances the generalizability of the ISI‐Bangla and supports its use in comparative studies and clinical assessments for both male and female patients. Previous studies have also established gender invariance, including in a Saudi nurse sample (Albougami and Manzar 2019) and among German adolescents, young adults, and adult workers (Gerber et al. 2016).

However, some divergence is notable when compared to studies that favored a unidimensional structure or three‐factor structure. For instance, a single‐factor model of the ISI was reported in a German sample (Gerber et al. 2016) and Swedish chronic pain patients (Dragioti et al. 2015). While a three‐factor structure was also reported in several studies (Castronovo et al. 2016; Chen et al. 2015; Fernandez‐Mendoza et al. 2012). These discrepancies may be attributed to sample characteristics, particularly clinical severity, comorbidity burden, or cultural interpretation of sleep disturbances, and highlight the importance of context‐specific validation. In this study, the distinct clustering of night and day symptoms may reflect the dual burden of chronic disease and insomnia, reinforcing the need to assess both domains independently for this population. The present CFA findings not only corroborate the ISI's theorized two‐factor structure but also validate its measurement equivalence across gender in a large Bangladeshi chronic disease sample, supporting its cross‐cultural and clinical applicability.

### 4.3 Rasch Analysis Findings in Context

The Rasch analysis of the ISI‐Bangla offers additional support for the scale's psychometric validity in a chronic disease population, revealing several important features concerning item functioning, dimensionality, and measurement precision. Consistent with earlier Rasch‐based investigations of the ISI, the present study found that the items functioned along a latent continuum of insomnia severity, with nighttime symptoms (items 1–4) clustering at the lower end of the difficulty scale and daytime impact items (items 5–7) showing higher item thresholds. This item ordering mirrors findings from previous studies, including Bangladeshi general population (Mamun et al. 2021) and Korean general population (Chung et al. 2024), confirming the ISI's ability to distinguish between gradations of symptom severity. Importantly, all items displayed satisfactory infit MnSq statistics, indicating acceptable item‐level conformity to model expectations. However, elevated outfit MnSq values for certain items (e.g., ISI_1, ISI_3, ISI_5) warrant attention. These findings echo previous studies (Bond 2015; Johansson et al. 2023), which caution that items with high outfit may reflect inconsistent responses from extreme scorers or content overlap, particularly in clinical populations with high symptom heterogeneity. While these misfits do not invalidate the scale, they highlight areas where refinements or expanded item banks might enhance precision in future adaptations.

The PCA of Rasch residuals and item loadings provided further support for the two‐factor dimensionality, with strong separation between night and day symptom clusters. These results align with bifactorial patterns previously identified in several studies (Al Maqbali et al. 2022; Albougami and Manzar 2019; Chung et al. 2024; Sadeghniiat‐Haghighi et al. 2014). The person reliability and separation indices indicated that the ISI‐Bangla can effectively distinguish two to three levels of insomnia severity in this population, especially for the night symptom subscale. Item reliability, however, varied—while excellent for night symptoms, it was low for the daytime impact subscale. This asymmetry may reflect restricted variability or skewed response patterns in items like ISI_5–ISI_7, especially given that daytime impairments may be underreported or confounded with chronic illness effects.

Lastly, while differential item functioning (DIF) by gender was modest and below the typical 0.50‐logit threshold (Johansson et al. 2023), several items, particularly ISI_3 to ISI_5, showed non‐negligible DIF. These findings suggest slight gender‐based variations in item interpretation, consistent with prior work showing that women may endorse certain sleep difficulties differently than men due to biological, social, or reporting factors (Ge et al. 2019; Taylor et al. 2007). Although these effects were small, they underscore the value of continued monitoring for measurement bias, especially in diverse and clinical populations. The Rasch analysis provides robust evidence for the ISI‐Bangla's construct validity while also identifying areas for potential item‐level refinement, particularly for daytime symptoms and gender‐related interpretive nuances.

### 4.4 Network Analysis Findings in Context

The network analysis of the ISI‐Bangla provided a nuanced, item‐level perspective on how insomnia symptoms interrelate, revealing a coherent yet multidimensional symptom structure consistent with both theoretical models and empirical evidence. The clustering of symptoms into two interconnected but distinguishable groups; nighttime symptoms (difficulty initiating and maintaining sleep) and daytime impacts (functional impairment and emotional distress), parallels the two‐factor structure supported by the CFA and Rasch analyses, underscoring the convergent validity of the ISI‐Bangla's dimensional representation.

Consistent with previous research, the strongest intra‐domain connection emerged between ISI_6 (“Noticeability of sleep problems”) and ISI_7 (“Worry/distress about current sleep problems”), reflecting a tight perceptual–emotional link. This finding replicates patterns observed in other studies (Bai et al. 2021; Chen et al. 2023), in which affective responses such as worry were strongly linked to perceived social or interpersonal consequences of insomnia. Such associations are clinically relevant, as distress about sleep is both a predictor and a perpetuator of chronic insomnia (Buysse 2013; Riemann et al. 2015). Within the nocturnal symptom domain, the triadic core comprising ISI_1 (“Difficulty falling asleep”), ISI_2 (“Difficulty staying asleep”), and ISI_3 (“Waking too early”) reflects foundational symptom clusters described in prior ISI network studies (Bai et al. 2021; Chen et al. 2023; Takano et al. 2023). The strong link between ISI_1 and ISI_2 suggests that initiation and maintenance difficulties are closely co‐occurring experiences, potentially stemming from shared etiological processes such as hyperarousal or poor sleep hygiene.

The identification of ISI_1 (“Difficulty falling asleep”) and ISI_6 (“Noticeability of sleep problems”) as the most central symptoms in the network indicates their influential roles in the overall insomnia symptom network. However, this pattern contrasts with findings from prior studies. For instance, a study of Chinese mental health professionals during the COVID‐19 pandemic reported “interference with daytime functioning” as the most central symptom (Bai et al. 2021), likely reflecting the heightened relevance of daytime impairments in high‐stress occupational settings. In contrast, “Difficulty staying asleep” was identified as the most central node in studies conducted among Japanese daytime workers (Takano et al. 2023) and caregivers of psychiatric inpatients during the COVID‐19 pandemic (Chen et al. 2023), suggesting that sleep continuity issues may be particularly salient in populations exposed to chronic caregiving burdens or structured daily routines. These differences highlight how central symptoms in insomnia networks may vary across sociocultural contexts, occupational demands, and pandemic‐related stress exposure, emphasizing the importance of population‐specific validation of psychometric structures.

Furthermore, ISI_4 (“Satisfaction with current sleep pattern”) emerged as the most important bridge symptom in our analysis across nearly all bridge centrality metrics. Its role as an integrative node suggests that dissatisfaction may be a perceptual construct linking objective symptoms (nighttime problems) to subjective evaluations and downstream functional impairments. In this context, ISI_4 appears to translate fragmented sleep experiences into a unified perception of poor sleep, thereby facilitating the propagation of distress into daytime functioning. Moreover, predictability estimates (*R* ^2^) indicated that ISI_6 (“Noticeability of sleep problems by others”) and ISI_7 (“Worry/distress about current sleep problems”) were highly accounted for by their immediate neighbors in the network. This suggests that these symptoms are likely downstream outcomes, potentially shaped by earlier or more primary symptoms such as sleep onset or maintenance difficulties. As such, while ISI_6 and ISI_7 may not initiate insomnia cascades, their centrality underscores their clinical importance as targets for alleviating emotional distress and perceived social consequences of sleep disturbances.

Last, the network invariance across gender reinforces the robustness and generalizability of the ISI‐Bangla structure. The absence of significant structural or global strength differences suggests that symptom interrelations do not differ markedly between male and female patients, aligning with prior research demonstrating consistent ISI network topology across demographic groups (Bai et al. 2021; Takano et al. 2023). This supports the use of ISI‐Bangla for both genders without necessitating different scoring or interpretive adjustments.

In sum, the network analysis provided a granular, visually interpretable, and theoretically coherent view of insomnia symptom interrelations. The alignment of network findings with factor analytic and Rasch‐based results bolsters the structural validity of the ISI‐Bangla and suggests its suitability for both clinical assessment and research use in chronic disease contexts.

## 5 Strength and Limitations

This study presents several notable strengths. It is among the first to comprehensively evaluate the psychometric properties of the Bangla version of the ISI among patients with chronic diseases using a triangulated analytic approach, including CFA, Rasch modeling, and psychological network analysis. The integration of these complementary frameworks provided robust evidence for the structural validity, reliability, and interpretability of the ISI‐Bangla in this population. The use of advanced item‐level analyses, including measurement invariance and bridge centrality, allowed for a nuanced understanding of symptom interrelations and their clinical salience. Additionally, the large and diverse sample recruited through face‐to‐face interviews enhances the ecological validity of the findings in low‐resource settings like Bangladesh. Furthermore, the reported analyses provide some degree of assurance that such psychometric properties will be confirmed when conducting similar assessments in our linguistic or cultural settings.

However, several limitations should be considered. First, the absence of a gold‐standard clinical diagnosis of insomnia (e.g., DSM‐5 or ICSD‐3 criteria confirmed by a sleep specialist) limits the ability to establish diagnostic cutoff scores or assess criterion validity. This limitation is common in community‐based or primary care studies conducted in LMICs, where access to specialist services is limited. Second, due to the cross‐sectional design, temporal and causal relationships between symptoms cannot be inferred. Longitudinal network analysis is needed to identify dynamic symptom changes over time or in response to interventions. Third, although a moderately large sample was used, subgroup‐specific analyses (e.g., by disease type or socioeconomic status) were underpowered and warrant future exploration. Fourth, a three‐factor CFA model was also tested and showed better global fit indices; however, this solution relied on a single‐indicator factor and produced very high inter‐factor correlations. For these reasons, it was not retained as the primary structure, and measurement invariance across gender was not pursued for this specification. Fifth, while network analysis offers novel insights into symptom connectivity and prioritization, the stability of centrality indices (e.g., node strength) was only moderate. Future studies should employ larger, more stratified samples and replicate findings across different clinical and cultural contexts. Last, the relatively low item reliability and separation in the daytime impact subscale of the Rasch model suggest the need for possible item refinement or expansion to capture a broader range of functional impairments experienced by patients with chronic illnesses.

Future research should validate these findings in longitudinal cohorts and explore the ISI‐Bangla's predictive validity regarding health outcomes such as treatment response, disease progression, and mental health comorbidities. The integration of objective sleep measures (e.g., actigraphy or polysomnography) and patient‐reported outcomes may enhance the validity and clinical utility of the ISI in Bangladeshi settings.

## 6 Conclusion

This study provides compelling psychometric evidence supporting the validity and reliability of the Bangla version of the ISI among patients with chronic diseases in Bangladesh. Through an integrative approach combining confirmatory factor analysis, Rasch modeling, and network analysis, the ISI‐Bangla was shown to possess a robust two‐factor structure distinguishing nighttime symptoms from daytime impairments. Although a three‐factor solution yielded somewhat improved global fit indices, it relied on a single‐indicator factor and exhibited high inter‐factor correlations, limiting its interpretability. Accordingly, the two‐factor model was retained as the most parsimonious and theoretically coherent structure. Rasch analysis confirmed good item‐level performance and dimensionality, though it flagged certain items for refinement. Network analysis further revealed key central and bridge symptoms, such as difficulty falling asleep, noticeability of sleep problems, and dissatisfaction with current sleep patterns, highlighting clinically actionable targets.

Importantly, measurement and network invariance across gender suggest that the ISI‐Bangla functions equivalently among male and female respondents, supporting its use in both clinical and research settings without gender‐specific scoring adjustments. The use of face‐to‐face data collection in a medically vulnerable population enhances the contextual relevance of the findings, contributing to culturally sensitive sleep assessment tools in low‐resource settings.

Together, these results affirm the ISI‐Bangla as a psychometrically sound instrument for assessing insomnia severity among chronic disease patients and underscore the value of multifaceted validation approaches. The insights generated from this study can inform targeted screening, symptom monitoring, and intervention strategies for insomnia in Bangladesh and similar contexts.

## Author Contributions

F.A.M., M.A.M., and M.A. contributed to the conceptualization of the study. Data curation was performed by F.A.M., M.A., and P.D. Formal analysis was conducted by FAM. Funding acquisition was managed by M.M.A. The investigation process involved F.A.M., M.A.M., M.A., and M.M.A. Methodology was developed collaboratively by F.A.M., M.A.M., and M.A., while project administration was overseen by M.A. and P.D. All authors provided necessary resources for the study. Supervision was carried out by M.M.A. and D.G. Validation and visualization were supported by F.A.M., M.A.M., M.M.A., and D.G. The original draft was written by F.A.M. and D.G., with all authors contributing to the review and editing of the manuscript.

## Funding

Dr. ALmerab is currently receiving funding support from the Princess Nourah Bint Abdulrahman University Researchers Supporting Project (Number PNURSP2025R563), Princess Nourah Bint Abdulrahman University, Riyadh, Saudi Arabia. DG is supported in part by National Institutes of Health grants HL166617 and HL169266.

## Conflicts of Interest

The authors declare no conflicts of interest

## Supporting information




**Supplementary Tables**: brb371040‐sup‐0001‐TableS1‐S5.docx

## Data Availability

The dataset associated with the manuscript is available from the corresponding author upon reasonable request.

## References

[brb371040-bib-0001] Albougami, A. , and M. D. Manzar . 2019. “Insomnia Severity Index: A Psychometric Investigation Among Saudi Nurses.” Sleep and Breathing 23, no. 3: 987–996. 10.1007/S11325-019-01812-8. 30850944

[brb371040-bib-0002] Al Maqbali, M. , N. Madkhali , and G. L. Dickens . 2022. “Psychometric Properties of the Insomnia Severity Index Among Arabic Chronic Diseases Patients.” SAGE Open Nursing 8: 23779608221107278. 10.1177/23779608221107278. 35769607 PMC9235306

[brb371040-bib-0003] Bai, W. , Y. Zhao , F. An , et al. 2021. “Network Analysis of Insomnia in Chinese Mental Health Professionals During the Covid‐19 Pandemic: A Cross‐Sectional Study.” Nature and Science of Sleep 13: 1921–1930. 10.2147/NSS.S326880. PMC8560171 34737660

[brb371040-bib-0004] Bastien, C. H. , A. Vallières , and C. M. Morin . 2001. “Validation of the Insomnia Severity Index as an Outcome Measure for Insomnia Research.” Sleep Medicine 2: 297–307. 10.1016/S1389-9457(00)00065-4. 11438246

[brb371040-bib-0005] Beaton, D. E. , C. Bombardier , F. Guillemin , and M. B. Ferraz . 2000. “Guidelines for the Process of Cross‐Cultural Adaptation of Self‐Report Measures.” Spine 25, no. 24: 3186–3191. 10.1097/00007632-200012150-00014. 11124735

[brb371040-bib-0006] Bond, T. 2015. *Applying the Rasch Model: Fundamental Measurement in the Human Sciences*. 3rd ed. Routledge. 10.4324/9781315814698.

[brb371040-bib-0007] Boone, W. J. 2016. “Rasch Analysis for Instrument Development: Why,When, and How?” CBE—Life Sciences Education 15, no. 4: ar48. 10.1187/CBE.16-04-0148. 27856555 PMC5132390

[brb371040-bib-0008] Borsboom, D. 2017. “A Network Theory of Mental Disorders.” World Psychiatry 16, no. 1: 5–13. 10.1002/WPS.20375. 28127906 PMC5269502

[brb371040-bib-0009] Brown, T. 2015. Confirmatory Factor Analysis for Applied Research. 2nd ed. Guilford Press.

[brb371040-bib-0010] Buysse, D. J. 2013. “Insomnia.” Jama 309: 706–716. 10.1001/JAMA.2013.193. 23423416 PMC3632369

[brb371040-bib-0011] Castronovo, V. , A. Galbiati , S. Marelli , et al. 2016. “Validation Study of the Italian Version of the Insomnia Severity Index (ISI).” Neurological Sciences 37: 1517–1524. 10.1007/S10072-016-2620-Z. 27234459

[brb371040-bib-0012] Chan, E. K. H. , B. D. Zumbo , W. Zhang , M. Y. Chen , I. Darmawanti , and O. P. Mulyana . 2014. “Validity and Validation in Social, Behavioral, and Health Sciences.” Validity and Validation in Social, Behavioral, and Health Sciences 54: 243–255. 10.1007/978-3-319-07794-9.

[brb371040-bib-0013] Chen, F. F. 2007. “Sensitivity of Goodness of Fit Indexes to Lack of Measurement Invariance.” Structural Equation Modeling 14, no. 3: 464–504. 10.1080/10705510701301834.

[brb371040-bib-0014] Chen, P. , Y. J. Zhao , F. R. An , et al. 2023. “Prevalence of Insomnia and Its Association With Quality of Life in Caregivers of Psychiatric Inpatients During the COVID‐19 Pandemic: A Network Analysis.” BMC Psychiatry [Electronic Resource] 23: 1–11. 10.1186/S12888-023-05194-W. 37964197 PMC10644468

[brb371040-bib-0015] Chen, P. Y. , C. M. Yang , and C. M. Morin . 2015. “Validating the Cross‐Cultural Factor Structure and Invariance Property of the Insomnia Severity Index: Evidence Based on Ordinal EFA and CFA.” Sleep Medicine 16, no. 5: 598–603. 10.1016/J.SLEEP.2014.11.016. 25863811

[brb371040-bib-0016] Cheung, G. W. , H. D. Cooper‐Thomas , R. S. Lau , and L. C. Wang . 2024. “Reporting Reliability, Convergent and Discriminant Validity With Structural Equation Modeling: A Review and Best‐practice Recommendations.” Asia Pacific Journal of Management 41: 745–783. 10.1007/S10490-023-09871-Y.

[brb371040-bib-0017] Christensen, K. B. , G. Makransky , and M. Horton . 2017. “Critical Values for Yen's Q3: Identification of Local Dependence in the Rasch Model Using Residual Correlations.” Applied Psychological Measurement 41, no. 3: 178–194. 10.1177/0146621616677520. 29881087 PMC5978551

[brb371040-bib-0018] Chung, S. , O. Ahmed , E. Cho , et al. 2024. “Psychometric Properties of the Insomnia Severity Index and Its Comparison With the Shortened Versions Among the General Population.” Psychiatry Investigation 21, no. 1: 9–17. 10.30773/PI.2023.0189. 38281736 PMC10822735

[brb371040-bib-0019] Dragioti, E. , T. Wiklund , P. Alföldi , and B. Gerdle . 2015. “The Swedish Version of the Insomnia Severity Index: Factor Structure Analysis and Psychometric Properties in Chronic Pain Patients.” Scandinavian Journal of Pain 9, no. 1: 22–27. 10.1016/J.SJPAIN.2015.06.001. 29911642

[brb371040-bib-0020] Epskamp, S. , D. Borsboom , and E. I. Fried . 2018. “Estimating Psychological Networks and Their Accuracy: A Tutorial Paper.” Behavior Research Methods 50: 195–212. 10.3758/S13428-017-0862-1. 28342071 PMC5809547

[brb371040-bib-0021] Epskamp, S. , A. O. J. Cramer , L. J. Waldorp , V. D. Schmittmann , and D. Borsboom . 2012. “qgraph: Network Visualizations of Relationships in Psychometric Data.” Journal of Statistical Software 48: 1–18. 10.18637/JSS.V048.I04.

[brb371040-bib-0022] Fernandez‐Mendoza, J. , A. Rodriguez‐Muñoz , A. Vela‐Bueno , et al. 2012. “The Spanish Version of the Insomnia Severity Index: a Confirmatory Factor Analysis.” Sleep Medicine 13: 207–210. 10.1016/J.SLEEP.2011.06.019. 22172961

[brb371040-bib-0023] Ge, L. , G. Guyatt , J. Tian , et al. 2019. “Insomnia and Risk of Mortality From All‐cause, Cardiovascular Disease, and Cancer: Systematic Review and Meta‐Analysis of Prospective Cohort Studies.” Sleep Medicine Reviews 48: 101215. 10.1016/J.SMRV.2019.101215. 31630016

[brb371040-bib-0024] Gerber, M. , C. Lang , S. Lemola , et al. 2016. “Validation of the German Version of the Insomnia Severity Index in Adolescents, Young Adults and Adult Workers: Results From Three Cross‐Sectional Studies.” BMC Psychiatry [Electronic Resource] 16: 1–14. 10.1186/S12888-016-0876-8. 27245844 PMC4888604

[brb371040-bib-0025] Haslbeck, J. M. B. , and L. J. Waldorp . 2020. “mgm: Estimating Time‐Varying Mixed Graphical Models in High‐Dimensional Data.” Journal of Statistical Software 93, no. 8: 1–46. 10.18637/JSS.V093.I08.

[brb371040-bib-0026] Hu, L. T. , and P. M. Bentler . 1999. “Cutoff Criteria for Fit Indexes in Covariance Structure Analysis: Conventional Criteria Versus New Alternatives.” Structural Equation Modeling 6, no. 1: 1–55. 10.1080/10705519909540118.

[brb371040-bib-0027] Johansson, M. , M. Preuter , S. Karlsson , M.‐L. Möllerberg , H. Svensson , and J. Melin . 2023. "Valid and Reliable? Basic and Expanded Recommendations for Psychometric Reporting and Quality Assessment." OSF Preprint, March 23. 10.31219/OSF.IO/3HTZC.

[brb371040-bib-0028] Jones, P. J. , R. Ma , and R. J. McNally . 2021. “Bridge Centrality: A Network Approach to Understanding Comorbidity.” Multivariate Behavioral Research 56, no. 2: 353–367. 10.1080/00273171.2019.1614898. 31179765

[brb371040-bib-0029] Jun, J. , C. G. Park , and M. C. Kapella . 2022. “Psychometric Properties of the Insomnia Severity Index for People With Chronic Obstructive Pulmonary Disease.” Sleep Medicine 95: 120–125. 10.1016/J.SLEEP.2022.04.017. 35569329

[brb371040-bib-0030] Li, C. H. 2016. “Confirmatory Factor Analysis With Ordinal Data: Comparing Robust Maximum Likelihood and Diagonally Weighted Least Squares.” Behavior Research Methods 48: 936–949. 10.3758/S13428-015-0619-7. 26174714

[brb371040-bib-0031] Lin, C. Y. , A. S. K. Cheng , B. Nejati , et al. 2020. “A Thorough Psychometric Comparison Between Athens Insomnia Scale and Insomnia Severity Index Among Patients With Advanced Cancer.” Journal of Sleep Research 29, no. 1: e12891. 10.1111/JSR.12891. 31328319

[brb371040-bib-0032] Linacre, J. M. 2002. “What Do Infit and Outfit, Mean‐square and Standardized Mean?” Rasch Measurement Transactions 16, no. 2: 878.

[brb371040-bib-0033] Mamun, M. A. , Z. Alimoradi , D. Gozal , et al. 2021. “Validating Insomnia Severity Index (ISI) in a Bangladeshi Population: Using Classical Test Theory and Rasch Analysis.” International Journal of Environmental Research and Public Health 2022, 19, no. 1: 225. 10.3390/IJERPH19010225. 35010485 PMC8750940

[brb371040-bib-0034] Manzar, M. D. , H. A. Jahrami , and A. S. Bahammam . 2021. “Structural Validity of the Insomnia Severity Index: A Systematic Review and Meta‐Analysis.” Sleep Medicine Reviews 60: 101531. 10.1016/J.SMRV.2021.101531. 34428679

[brb371040-bib-0035] Masters, G. N. 1982. “A Rasch Model for Partial Credit Scoring.” Psychometrika 47: 149–174. 10.1007/BF02296272.

[brb371040-bib-0036] Morin, C. M. , G. Belleville , L. Bélanger , and H. Ivers . 2011. “The Insomnia Severity Index: Psychometric Indicators to Detect Insomnia Cases and Evaluate Treatment Response.” Sleep 34, no. 5: 601–608. 10.1093/SLEEP/34.5.601. 21532953 PMC3079939

[brb371040-bib-0037] Quan, Y. , and C. Wang . 2025. “Collapsing or Not? A Practical Guide to Handling Sparse Responses for Polytomous Items.” Methodology 21, no. 1: 46–73. 10.5964/METH.14303.

[brb371040-bib-0038] Riemann, D. , C. Nissen , L. Palagini , A. Otte , M. L. Perlis , and K. Spiegelhalder . 2015. “The Neurobiology, Investigation, and Treatment of Chronic Insomnia.” Lancet Neurology 14, no. 5: 547–558. 10.1016/S1474-4422(15)00021-6. 25895933

[brb371040-bib-0039] Roth, T. 2007. “Insomnia: Definition, Prevalence, Etiology, and Consequences.” Journal of Clinical Sleep Medicine 3: no. 5 supplement: S7–S10. 10.5664/jcsm.26929. 17824495 PMC1978319

[brb371040-bib-0040] Sadeghniiat‐Haghighi, K. , A. Montazeri , A. Khajeh‐Mehrizi , S. Nedjat , and O. Aminian . 2014. “The Insomnia Severity Index: Cross‐Cultural Adaptation and Psychometric Evaluation of a Persian Version.” Quality of Life Research 23: 533–537. 10.1007/S11136-013-0489-3. 23912857

[brb371040-bib-0041] Takano, Y. , R. Ibata , N. Nakano , and Y. Sakano . 2023. “Network Analysis to Estimate Central Insomnia Symptoms Among Daytime Workers At‐risk for Insomnia.” Scientific Reports 13: 1–8. 10.1038/S41598-023-43802-7. 37775548 PMC10542340

[brb371040-bib-0042] Taylor, D. J. , L. J. Mallory , K. L. Lichstein , H. H. Durrence , B. W. Riedel , and A. J. Bush . 2007. “Comorbidity of Chronic Insomnia With Medical Problems.” Sleep 30, no. 2: 213–218. 10.1093/SLEEP/30.2.213. 17326547

[brb371040-bib-0043] van Borkulo, C. D. , R. van Bork , L. Boschloo , et al. 2022. “Comparing Network Structures on Three Aspects: A Permutation Test.” Psychological Methods 28, no. 6: 1273–1285. 10.1037/MET0000476. 35404628

[brb371040-bib-0044] Xia, Y. , and Y. Yang . 2019. “RMSEA, CFI, and TLI in Structural Equation Modeling With Ordered Categorical Data: The Story They Tell Depends on the Estimation Methods.” Behavior Research Methods 51: 409–428. 10.3758/S13428-018-1055-2. 29869222

[brb371040-bib-0045] Yen, W. M. 1993. “Scaling Performance Assessments: Strategies for Managing Local Item Dependence.” Journal of Educational Measurement 30: 187–213. 10.1111/j.1745-3984.1993.tb00423.x.

