# Supplementary material for: Psychometric Evaluation of the Insomnia Severity Index in Chronic Disease Patients Using Three Complementary Approaches
Source: Brain Behav. 2025 Nov 10;15(11):e71040. doi: 10.1002/brb3.71040 (PMC12602257; doi:10.1002/brb3.71040)
Supplement: Supplementary file 1 — Supplementary Tables: brb371040‐sup‐0001‐TableS1‐S5.docx [file BRB3-15-e71040-s001.docx]

**Supplementary material for**

**Psychometric Evaluation of the Insomnia Severity Index Structure in Chronic Disease Patients Using 3 Complementary Approaches**

Firoj Al-Mamun^1,2*^, Mohammed A Mamun^1,2^, Mohamad Arif ^1,3^, Pronab Das ^1,4^, Moneerah Mohammad Almerab^5^, David Gozal^6*^

1. CHINTA Research Bangladesh, Savar, Dhaka, Bangladesh [FAM: [firojphiju@gmail.com](mailto:firojphiju@gmail.com)]
2. Department of Public Health, University of South Asia, Dhaka, Bangladesh [MAM: [mamunphi46@gmail.com](mailto:mamunphi46@gmail.com)]
3. Department of Ayurvedic Medicine, Institute of Teaching and Research in Ayurveda, Jamnagar, Gujarat, India [MA: [m.arif@thechinta.org](mailto:m.arif@thechinta.org)]
4. One Health Institute, Chattogram Veterinary and Animal Sciences University, Chattogram 4225, Bangladesh [PD: [pronab@thechinta.org](mailto:pronab@thechinta.org)]
5. Department of Psychology, College of Education and Human Development, Princess Nourah Bint Abdulrahman University, Riyadh, Saudi Arabia (MMA: [mmalmreab@pnu.edu.sa](mailto:mmalmreab@pnu.edu.sa))
6. Departments of Pediatrics and Biomedical Sciences and Office of the Dean, Joan C. Edwards School of Medicine, Marshall University, Huntington, West Virginia, USA [DG: [gozal@marshall.edu](mailto:gozal@marshall.edu)]

Correspondence: David Gozal, Joan C. Edwards School of Medicine, Marshall University, Huntington, West Virginia, USA; Email: [gozal@marshall.edu](mailto:gozal@marshall.edu);

Firoj Al-Mamun, Department of Public Health, University of South Asia, Dhaka, Bangladesh; Email: [firojphiju@gmail.com](mailto:firojphiju@gmail.com)

**Supplementary Table S1**. Item-level and reliability analysis of the ISI-Bangla (three-factor solution)

| ISI Item | Latent Factor | Standardized Loading (CFA) | AVE | CR | α / αₒᵣ𝒹 | ω |
| --- | --- | --- | --- | --- | --- | --- |
| ISI_1 – Difficulty falling asleep | Night Symptom | 0.841 | 0.788 | 0.917 | 0.903 / 0.937 | 0.851 |
| ISI_2 – Difficulty staying asleep | Night Symptom | 0.861 |  |  |  |  |
| ISI_3 – Problem waking up too early | Night Symptom | 0.957 |  |  |  |  |
| ISI_4 – Satisfaction with current sleep pattern | **Satisfaction** | 1.000 | **1.000** | **1.000** | — | — |
| ISI_5 – Interference with daily functioning | Daytime Distress | 0.835 | 0.830 | 0.936 | 0.889 / 0.929 | 0.913 |
| ISI_6 – Noticeability of sleep problems by others | Daytime Distress | 0.953 |  |  |  |  |
| ISI_7 – Worry/distress about current sleep problem | Daytime Distress | 0.939 |  |  |  |  |
| ****Note.**** CFA = Confirmatory factor analysis; AVE = Average variance extracted; CR = Composite reliability; α = Cronbach’s alpha assumes continuous indicators; αₒᵣ𝒹 = Ordinal alpha based on polychoric correlations, recommended for ordinal data; ω = McDonald’s omega. ****AVE, CR, α, αₒᵣ𝒹, and ω are reported at the factor level.**** The ****Satisfaction factor (ISI_4)**** is a single-indicator latent construct identified by fixing its factor loading to 1.0 and residual variance to zero. This yields artificially perfect AVE and CR values; reliability indices are not meaningful for this factor and are therefore not reported. | | | | | | |

**Supplementary Table S2.** Residual matrix (Q3) for ISI items

|  | Night time symptoms | | | |
| --- | --- | --- | --- | --- |
|  | ISI_1 | ISI_2 | ISI_3 | ISI_4 |
| ISI_1 | 1.00 | 0.61 | 0.52 | 0.22 |
| ISI_2 | 0.61 | 1.00 | 0.56 | 0.20 |
| ISI_3 | 0.52 | 0.56 | 1.00 | 0.30 |
| ISI_4 | 0.22 | 0.20 | 0.30 | 1.00 |
| Daytime impact symptoms | | | | |
|  | ISI_5 | ISI_6 | ISI_7 |  |
| ISI_5 | 1.00 | 0.43 | 0.29 |  |
| ISI_6 | 0.43 | 1.00 | 0.50 |  |
| ISI_7 | 0.29 | 0.50 | 1.00 |  |

**Supplementary Table S3.** Edge weight matrix for the ISI Bangla network structure

|  | ISI_1 | ISI_2 | ISI_3 | ISI_4 | ISI_5 | ISI_6 | ISI_7 |
| --- | --- | --- | --- | --- | --- | --- | --- |
| ISI_1 | 0.0000 | 0.5005 | 0.2612 | 0.1652 | -0.1218 | 0.0000 | 0.0911 |
| ISI_2 | 0.5005 | 0.0000 | 0.3600 | 0.0838 | -0.0392 | 0.0256 | 0.0140 |
| ISI_3 | 0.2612 | 0.3600 | 0.0000 | 0.1956 | 0.0000 | 0.1193 | 0.0693 |
| ISI_4 | 0.1652 | 0.0838 | 0.1956 | 0.0000 | 0.3051 | 0.0438 | 0.1406 |
| ISI_5 | -0.1218 | -0.0392 | 0.0000 | 0.3051 | 0.0000 | 0.4387 | 0.1447 |
| ISI_6 | 0.0000 | 0.0256 | 0.1193 | 0.0438 | 0.4387 | 0.0000 | 0.5086 |
| ISI_7 | 0.0911 | 0.0140 | 0.0693 | 0.1406 | 0.1447 | 0.5086 | 0.0000 |

**Supplementary Table S4.** Centrality metrics and predictability for the ISI items

| Node | Strength | Closeness | Betweenness | Predictability (R²) |
| --- | --- | --- | --- | --- |
| ISI_1 | 1.140 | 0.0241 | 4 | 0.726 |
| ISI_2 | 1.023 | 0.0213 | 0 | 0.723 |
| ISI_3 | 1.005 | 0.0257 | 4 | 0.714 |
| ISI_4 | 0.934 | 0.0286 | 2 | 0.727 |
| ISI_5 | 1.049 | 0.0273 | 4 | 0.722 |
| ISI_6 | 1.136 | 0.0251 | 4 | 0.755 |
| ISI_7 | 0.968 | 0.0210 | 0 | 0.771 |

**Supplementary Table S5.** Bridge centrality metrics for each ISI symptom node across two communities

| Node | Community | Bridge Strength | Bridge Betweenness | Bridge Closeness | Bridge Expected Influence (1-step) | Bridge Expected Influence (2-step) |
| --- | --- | --- | --- | --- | --- | --- |
| ISI_1 | 1 | 0.304 | 1 | 0.0978 | 0.1799 | 0.4304 |
| ISI_2 | 1 | 0.171 | 0 | 0.0859 | 0.1209 | 0.4104 |
| ISI_3 | 1 | 0.406 | 3 | 0.1119 | 0.4061 | 0.7592 |
| ISI_4 | 2 | 0.428 | 3 | 0.1478 | 0.4282 | 0.7415 |
| ISI_5 | 2 | 0.113 | 0 | 0.0917 | -0.0612 | 0.0798 |
| ISI_6 | 2 | 0.144 | 0 | 0.0825 | 0.1441 | 0.3424 |
| ISI_7 | 2 | 0.196 | 1 | 0.0885 | 0.1958 | 0.4363 |
